# Effects of Combined Visual-Motor Response Training on Cognitive Function and Brain Plasticity Mechanisms in Various Populations: Protocol for a Single-Center, Open-Label, Controlled Clinical Trial

**DOI:** 10.2196/56424

**Published:** 2024-10-07

**Authors:** Wenlong Yu, Jiamin Gao, Ping Zhu

**Affiliations:** 1 Department of Orthopaedics Longhua Hospital Shanghai University of Traditional Chinese Medicine Shanghai China; 2 Shanghai University of Traditional Chinese Medicine Shanghai China

**Keywords:** visual-motor, response ability training, cognitive function, brain plasticity mechanism, study protocol

## Abstract

**Background:**

Cognitive impairment is one of the major diseases facing the aging population. The progressive decline of cognitive function can lead to declining health or even the loss of life, work, and social ability. Exercise and behavioral stimulation can increase neurotransmitters in the brain and improve overall health and cognitive function. Reactivity training can mobilize neuromuscular function and induce changes in brain plasticity, which may effectively improve cognitive dysfunction and delay the occurrence and development of Alzheimer disease; however, the evidence supporting its effectiveness is still limited.

**Objective:**

This study aims to explore the effectiveness and reliability of visual-motor reaction training in improving cognitive function, thereby promoting the application of novel nonpharmacological therapies.

**Methods:**

This study is a single-center, open-label, controlled clinical trial. A total of 78 participants will be recruited for the study, including an equal number of athletes, ordinary healthy college students, and ordinary older adults in the community. Participants will receive 2 weeks of visual-motor response training. The primary outcome of this study is to assess differences in functional magnetic resonance imaging (fMRI) at 2 weeks. The secondary outcomes were the following: acousto-optic response time, Hamilton Depression Rating Scale (HAM-D), Hamilton Anxiety Rating Scale (HAM-A), Mini Mental State Examination (MMSE), Activity of Daily Living (ADL) Scale, Subjective Cognitive Decline Questionnaire–9 (SCD-Q9), a 10-word memory test, and safety.

**Results:**

The study was approved by the Shanghai Clinical Research Ethics Committee on January 2, 2024 (SECCR/2023-162-01). As of September 11, 2024, we have completed the recruitment of all 3 groups of volunteers. We expect to complete data collection and analysis by February 2025.

**Conclusions:**

The purpose of this study is to compare improvements in brain perceptual motor functions and cognitive levels across different populations through response ability training and to explore the efficacy and safety of exercise-based nonpharmacological therapies in improving cognitive function. Other potential benefits include understanding the functional differences and perceptual characteristics of the brain’s perceptual-motor system between athletes and the general population and exploring the adaptability of the brain in acquiring skills during competitive sports training. This could provide an evidence base for early sports talent development and broader youth development.

**Trial Registration:**

Chinese Clinical Trial Registry ChiCTR2400079602; https://tinyurl.com/23fbbndw

**International Registered Report Identifier (IRRID):**

DERR1-10.2196/56424

## Introduction

Population aging has become an increasingly serious global problem. In recent years, the global older population has increased rapidly. It is estimated that by around 2050, the number of people aged 60 and older will reach 2 billion, accounting for more than 21% of the world’s population [1]. Older people have a high prevalence of cognitive impairment. China has the largest older population in the world, and cognitive impairment has a significant social impact on demographic imbalance [2]. Research shows that there are 15.07 million people with dementia aged 60 and older in China, including 9.83 million with Alzheimer disease, 3.92 million with vascular dementia, and 1.32 million with dementia [3]. With the accelerated aging of Chinese society, the increase of brain diseases, such as stroke, Parkinson disease, and Alzheimer disease, has increased cognitive dysfunction in the population; this seriously affects national health and greatly increases the economic and mental burden on patients, their family members, and society [4]. The demand for cognitive function training and cognitive impairment prevention in families and society is increasing daily, so the search for simple and easy-to-implement cognitive ability training methods is of great scientific significance and practical value.

Extensive studies on human beings have shown that exercise and response training are beneficial to overall health and cognitive function, especially in the aging population. In 1979, the United States Public Health Service detailed the role of exercise in health promotion, and for the first time, elevated national physical activity to the level of the national health plan, making exercise and health promotion a research hot spot. Exercise helps to improve the health of older people, enhances physical functioning, and reduces the incidence of chronic diseases and disabilities [5]. Studies have shown that exercise and behavioral stimulation can increase neurotransmitters in the brain, decrease levels of oxidative stress and inflammation, increase cerebral blood flow, and modulate the hypothalamic-pituitary-adrenal axis, which in turn alters the prefrontal cortex and the temporal lobe, increasing the volume of the hippocampal gyrus [6-8]. This change can promote neural activation in the cerebral cortex, enhance synaptic plasticity, and, it is hoped, improve cognitive ability and motor coordination [9].

In addition, response training is of great significance in ameliorating cognitive impairment and delaying the occurrence of dementia and Alzheimer disease [10]. People with different physical abilities may receive different results from reactivity training. Previous studies have found that the plasticity of the brain can change, particularly among professional athletes who have received extensive physical training [11-13]. In particular, volleyball players performed better in the implementation of control tasks and visual-spatial attention processing tasks. The action observation network plays an important role in these tasks, with the upper parietal lobe and auxiliary motor area being positively correlated with anticipatory capacity [14,15].

However, most forms of exercise are not suitable for the older population due to physical limitations. Therefore, the design and development of a combination of muscle and balance function training is an effective way to improve muscle coordination, prevent falls, and improve the cognitive state of older people. With advancements in technology, cognitive training equipment used in the community or by families is being widely used, which can better meet the issues of accessibility and portability of cognitive training. This study uses smart equipment to train the visual-motor response abilities of volleyball players, ordinary college students, and older people. We use functional magnetic resonance imaging (fMRI) to visualize the characteristic brain regions involved in perception of movement across different physical functions and explore the effects of cognitive training on cognitive function and the mechanisms of brain plasticity. The aims of this study are as follows: (1) to compare the functional advantages and characteristics of the brain perceptual-motor system in volleyball players and ordinary younger and older people; (2) to explore the influence of visual-motor response ability training on cognitive function in people with different physical functions; and (3) to preliminarily confirm the efficacy and safety of exercise-based nondrug therapy in improving cognitive function.

## Methods

### Study Design

**Figure 1:**
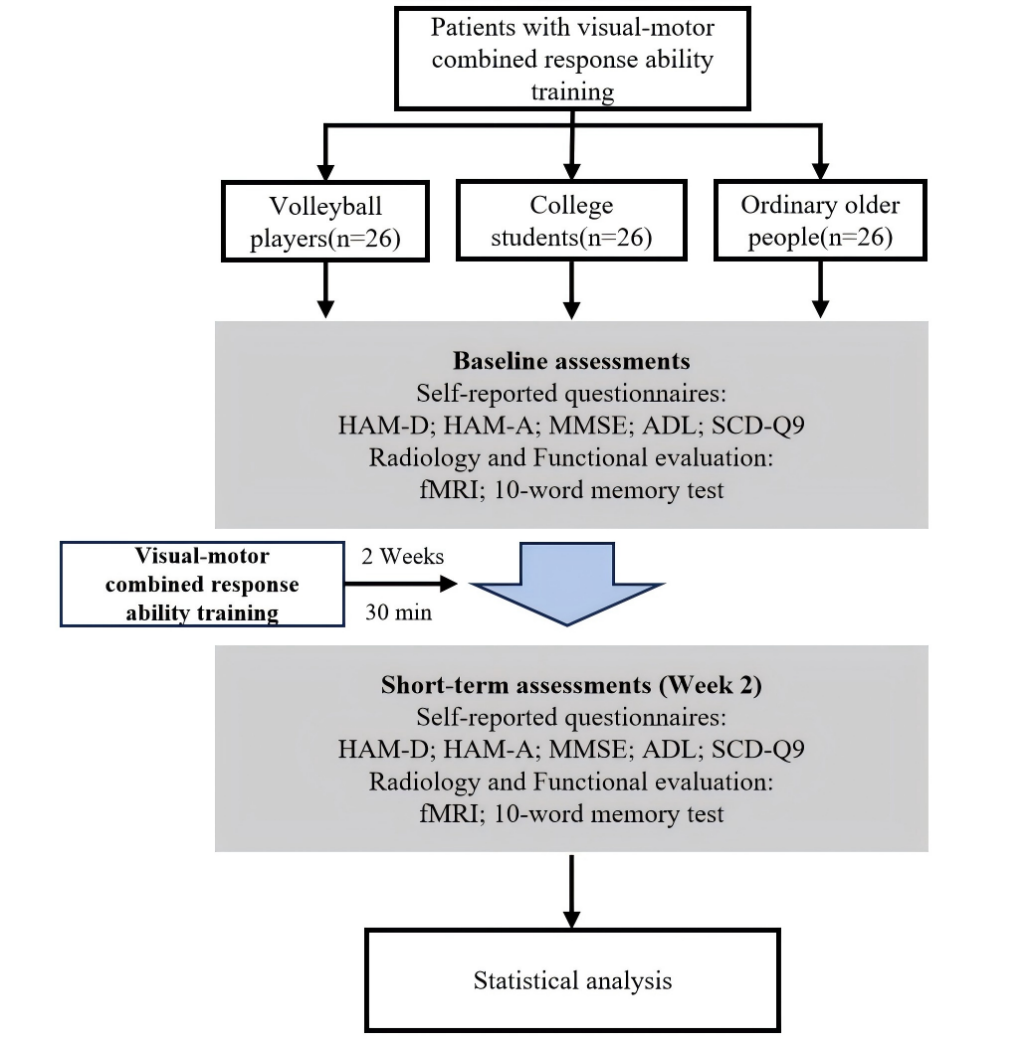
Study flowchart. ADL: Activity of Daily Living; fMRI: functional magnetic resonance imaging; HAM-A: Hamilton Anxiety Rating Scale; HAM-D: Hamilton Depression Rating Scale; MMSE: Mini Mental State Examination; SCD-Q9: Subjective Cognitive Decline Questionnaire–9.

This is a prospective, single-center, open-label, controlled clinical trial conducted between January 2022 and December 2025. This study will recruit professional volleyball players, ordinary college students, and older people without any professional sports training. In total, 78 participants will be recruited at the Shanghai University of Traditional Chinese Medicine, China, via the university website or posters, with 26 participants in each group. All participants will undergo baseline assessments, including acousto-optic response time tests and fMRI scans. This will be followed by 2 weeks of visual-motor response training. After 2 weeks, the outcomes of the intervention will be evaluated (Figure 1).

### Participants

The participants in the athlete group will be recruited from the high-level volleyball team of Shanghai. The college students will be recruited from the Shanghai University of Traditional Chinese Medicine through posters on campus and advertisements in campus media. Participants in the older population group will be recruited from healthy older adults in the community; in-person outreach will be conducted in randomly selected neighborhoods in Shanghai to attract active participants. Within each group, an equally spaced sampling method will be used to determine the final participants in the study. Participants need to understand and agree to this study, voluntarily undergo preintervention assessment and 2 weeks of visual-motor combination response training, and sign an informed consent form.

### Sample Size

Because of the exploratory nature of this study, it is impossible to estimate the total number of cases. Based on previous data, fMRI studies can be performed in groups of 12 participants with adequate statistical efficacy [16]. For the planned number of 20 participants in each group, if a 1-way ANOVA is used, it will be possible to detect a standardized mean-value effect (Cohen *d*) of 0.4, with a power of 80% and a significance level of 5% [17,18]. Assuming a loss to follow-up of 20%, the total sample size required for the study is 78 (n=26 in each group).

### Inclusion Criteria

The fulfillment of the following conditions is a prerequisite for all participants: (1) meet the “Technical Grade Standard of Volleyball Players” issued by the General Administration of Sports of China and obtain a grade II title or higher; (2) vision or corrected vision of 1.0; (3) no history of mental illness, brain trauma, and other diseases that affect brain structure and function; (4) meet fMRI scanning conditions, such as not having metal implants in the body or claustrophobia; and (5) being right-handed. Volleyball players should be aged 18 to 23 years; college students should be aged 18 to 23 years and have no professional sports training; older adults should be aged 55 to 70 years, have no professional sports training, and have a physical condition that meets the basic research needs (including self-care and normal movement of limbs and joints).

### Exclusion Criteria

Participants meeting any one of the following criteria will be excluded from this trial: (1) serious diseases of the heart and lung system; (2) diagnosis of mental illness, severe depression (suicidal tendency), or epilepsy; (3) metabolic systemic diseases, such as diabetes and uremia; (4) skeletal and muscle diseases, such as fractures, ligament injuries, severe arthritis, meniscus injuries, or spinal injuries; (5) hearing or visual impairment; and (6) use of drugs (eg, antiepileptic drugs, sedative drugs, and hypnotic drugs) that change the excitability of the cerebral cortex in the past 3 months.

### Dropout Criteria

Dropout criteria for patients are as follows: (1) withdrawal from the study and (2) incomplete implementation of the treatment plan.

### Suspension Criteria

If a patient develops other diseases or experiences serious adverse reactions during treatment, the trial will be discontinued by mutual decision of the participant and investigators.

### Intervention

The implementation team of the intervention process includes a sports rehabilitation physician, a neurologist, a volleyball coach, a graduate student, and 2 undergraduate students. Before the formal intervention, the researchers were made familiar with the intervention equipment and intervention process through training. A between-group control design will be used with the same interventions. The 3 groups of participants will receive the same visual-motor response training for 30 minutes a day over 14 days. Response training will be completed with the help of gForceDual equipment and Neuro Rehab System (OYMotion). The participants download the Neuro Rehab System–based app and connect the gForceDual device to their mobile device through Bluetooth. During each training session, the gForceDual equipment will be secured to the transverse striation of the distal wrist of the participants’ right upper limb, and its electrode will be attached to the ventral part of the palmar longus muscle, brachioradialis muscle, radial carpal flexor muscle, and ulnar carpal flexor muscle. The response sensitivity will be adjusted via the app, and the participants will be guided by visual information to carry out wrist dorsal extension and flexion. The speed adjustment function in the app will be set to divide the training speed into gears, with a total of 10 gears. The default setting is sixth gear during the intervention. If an older person cannot keep up with the response, the app will downshift through gears; conversely, if an athlete can adapt well to a high gear, the app will upshift the gear. The researchers will record electromyographic signals produced by dorsal extension and flexion of the wrist.

### Outcome Measures

The aim of the study is to explore the functional characteristics of brain perceptions of movement and the cognitive changes in different groups after response ability training. The primary outcome is defined by differences in fMRI images of characteristic areas of the brain before and after the intervention. A Siemens Magnetom Verio 3.0T MRI system will be used for scanning before and after the intervention.

The secondary outcomes are as follows: acousto-optic response time, Hamilton Depression Rating Scale (HAM-D) [19], Hamilton Anxiety Rating Scale (HAM-A) [20], Mini Mental State Examination (MMSE) [21], Activity of Daily Living (ADL) Scale [22], Subjective Cognitive Decline Questionnaire–9 (SCD-Q9) [23], a 10-word memory test, and safety. At the beginning and the end of the intervention, all participants will be asked to fill in the test questionnaire with the assistance of the researchers. The EP 204 audio-visual reaction time measuring instrument (produced by the Science and Education Instrument Factory of East China Normal University) is used for acousto-optic response time. The instrument automatically presents auditory and visual (simultaneous 4-color light) stimuli. In each experiment, the number of light and sound stimuli is equal, and if the test is set to be repeated 20 times, then each stimulus will be presented 10 times in a random order. When the participant hears (or sees) the stimuli, they must lift their finger from the circular hole on the response keyboard. The instrument automatically records the time from the presentation of the stimulus to the time the participants lifts their finger from the circular hole on the response keyboard; this is defined as the reaction time for the sound (or light). During the experiment, if the participant does not put their finger back into the circular hole on the response keyboard, the instrument will automatically enter a waiting state until the participant puts their finger back and the next test can be performed. The 10-word memory test uses PowerPoint slides to have the participant read 10 words, presenting each word for 2 seconds. The participants will be then asked to recall the words out loud. The evaluator will check each correctly recalled word. A total of 3 reading and recall trials will be administered. The score will be the average number of words not correctly recalled over the 3 trials. HAM-D, HAM-A, MMSE, and SCD-Q9 are all scale-assessed and uniformly evaluated, and they will be used to assess a participant’s psychological state. The ADL Scale will be used for participant baseline condition screening and assessment of the degree of improvement in mobility after treatment.

Adverse events are defined as any adverse experience that may be related to the trial treatment procedure that occurs during the course of the study. Any discomfort reported by the participants during the study will be carefully recorded.

### Safety Measures

An excessively high gear or initial discomfort may cause dizziness, nausea, or fatigue in some participants during the audio-visual response training. During the training, research assistants will monitor the participants’ status and provide medical assistance as needed. If a participant is unable to adapt to our training model, the training session will be promptly terminated, and follow-up medical counseling and assistance will be offered.

### Statistical Analysis

All variables that can be measured directly will be analyzed descriptively by tabulating the empirical distributions. For continuous variables, mean (with SD), median, minimum, and maximum values will be documented. Classification variables will be recorded as incidence and percentage. Measurement data following a normal distribution will be analyzed with a 2-way repeated measures ANOVA. For intergroup factors, the participants will be divided into 3 groups, comprising athletes, young people, and older people. The intragroup factor will be evaluation time, which has 2 levels—before and after the intervention. The main effects and interactions among the 3 groups will be analyzed. For nonnormally distributed data, the Wilcoxon symbolic rank test will be used for intragroup analysis, and the Mann-Whitney *U* test will be used for intergroup comparison. The significance level of the statistical test is set at α=.05, and bilateral tests will be carried out. Differences in classification variables will be calculated using the *χ*^2^ test. *P*<.05 will be considered statistically significant.

The differences in brain fMRI images will be analyzed by MATLAB (R2014a; MathWorks) and Statistical Parametric Mapping (version 12; UCL Queen Square Institute of Neurology) [24]. Based on the intergroup analysis of the resting-state amplitude of low-frequency fluctuations (ALFF) and regional homogeneity (ReHo) indices, we will perform a flexible-design ANOVA to characterize the individual samples at each time point. Analyses will include time main effects, grouping main effects, time × grouping interactions, and statistical parameter diagrams. The post hoc test mask will reflect differences in brain regions where *P*<.01 and voxel >10, as determined by the ANOVA results. The intergroup analysis of functional connections will be done using GRETNA (Neuroimaging Tools and Resources Collaboratory) [25]. The functional connection differences among the 3 groups will be analyzed with a 2-factor repeated measurement ANOVA, and the intragroup analysis will be conducted with a paired 2-tailed *t* test. A *P* value <.01 is considered to show statistical significance. BrainNet Viewer (Neuroimaging Tools and Resources Collaboratory) will be used to draw schematic maps of brain regions with significant differences.

### Quality Control

All the researchers in this trial will receive a 5-day training course. Researchers involved in the treatment process will not be allowed to perform statistical analysis of the data. Data entry and statistical analysis will be the responsibility of professional researchers, and these participants will be unaware of the specific sources of data and the intervention process. The trial will establish an independent data monitoring committee, composed of 5 members, who will not participate in the operation of the trial and have no conflict of interest with the study. Members of the data monitoring committee will regularly review the research data to ensure the safety of participants and the reliability of the research data.

### Ethical Considerations

The study will be conducted in accordance with the principles of the Helsinki Declaration and was approved by the review and ethics committee of the Shanghai Ethics Committee for Clinical Research on January 2, 2024 (ID: SECCR/2023-162-01). The study protocol has been registered on ClinicalTrials.gov and the Chinese Clinical Trial Registry (ID: ChiCTR2400079602). All participants will be aware of the potential risks and benefits of the study. All participants will provide informed consent and can opt out at any point during the study process. Use of participants’ personal data will be prohibited for other purposes, and the data will be stored in specific cabinets. Treatment and medical advice will be provided at no cost to participants who experience accidental injuries in this study.

### Dissemination of the Study Data

At the end of the study, a summary of the results will be provided to the participants. The results of the study will be published in peer-reviewed journals and presented at national and international conferences.

### Availability of Data and Materials

The research data will be assigned a numeric code that can only be assessed by the specified researcher. The full dataset that can be used and analyzed at the end of the study can be accessed by contacting the corresponding author.

## Results

As of September 11, 2024, we have completed the recruitment of all 3 groups of volunteers. We expect to complete data collection and analysis by February 2025.

## Discussion

### Principal Findings

This paper presents the protocol for a single-center, open-label, controlled clinical trial that will evaluate the effects of combined visual-motor response training on cognitive function to help patients recover and maintain quality of life. We aim to directly test the hypotheses that the perceptual-motor system of the brain of people with long-term exposure to combined visual-motor response ability training has a functional advantage compared to the general population and that combined visual-motor response ability training is effective in improving cognitive functioning in both the general population and the aging population. In addition, the trial will evaluate the effectiveness and safety of exercise-based nonpharmacological therapies when applied to improve cognitive function. This study highlights the importance of finding novel therapies for cognitively impaired populations. The implementation of exercise-based, nonpharmacological therapies is a potential option with profound implications for managing chronic diseases and improving health care in an aging society.

Advanced cognitive functions, such as cognitive flexibility, working memory, and long-term memory, tend to decline with age after the age of 20 [26]. Cognitive aging is usually accompanied by atrophy in brain structures such as the caudate nucleus, cerebellar hemisphere, lateral prefrontal cortex, and hippocampal gyrus [27]. The results of several randomized controlled trials have shown that combined exercise and biofeedback-based training are effective in ameliorating cognitive impairment and improving physical functioning, memory, and executive function [28-31]. However, Griffen et al [32] confirmed through a randomized controlled trial that resistance exercise does not significantly improve cognitive function [32]. Other studies have had similar results, indicating that while exercise may improve overall cognitive functioning, it primarily enhances executive ability, with minimal impact on memory and social cognition [33,34]. Unlike the general population and the older population, improvements in cognitive functioning have long been demonstrated in athletes undergoing combined exercise and biofeedback training [35-37]. Therefore, differential findings prompted us to explore the effectiveness of visual-motor response training.

fMRI locates complex cognitive activities in the brain with high spatial resolution, providing a more accurate and objective view of brain information processing during exercise and identifying the brain regions involved in effective and reasonable information processing. ALFF is a relatively stable metric in fMRI sequences that responds to the low-frequency electrical activity of local neurons in the resting state [38]. ALFF and ReHo are used to identify neural networks throughout the brain [39]. Based on the assumption that a voxel is similar to its neighboring voxels over time, neural networks can be identified by evaluating and comparing the synchronization or similarity over time between each voxel in the region of interest and its neighboring voxels [39,40]. Unlike previous studies, we will not only use questionnaires and scales to assess the cognitive function of patients but also assess the changes in the functional structure of the brain by fMRI, which will make the evidence of the study more explicit. This exploratory study will attempt to open new horizons for subsequent trials. If the hypothesis is valid, combined visual-motor response ability training will replace exercise-only interventions as a new preventive measure and treatment method, which is significant for the current society with an aging population.

### Strengths and Limitations

The study has some strengths. First, it used visual-motor response training rather than an exercise-only intervention, aiming to increase biofeedback and identify more effective measures for improving cognitive function. Second, we intervened with 3 groups with different training bases, physical functioning, and cognitive functioning to provide a broader perspective on the effectiveness of the intervention. In addition, this study simultaneously assessed changes in psychological status and functional brain imaging, providing a broader evidence base for the efficacy of the intervention. However, the study has some limitations. First, the study’s intervention period is short, and it will not be known whether the intervention effect is significant over a 2-week period. The lack of randomized assignment in the study is also a limitation, as it can potentially affect the accuracy of the findings. Therefore, further studies with high evidence quality should be conducted in the future to better explore the efficacy of combined visual-motor response ability training.

### Conclusions

This protocol outlines the design of a single-center, open-label, controlled clinical trial that evaluates the effects of a 2-week intervention with combined visual-motor response ability training on differences in characteristic brain regions in fMRI images, cognitive functioning, physical functioning, and social and psychological indicators. By examining the clinical effects of combined visual-motor response ability training, this study may provide an effective physical therapy for preventing cognitive decline, which is important for health care effectiveness.

## Abbreviations

ADL: Activity of Daily Living
ALFF: amplitude of low-frequency fluctuations
fMRI: functional magnetic resonance imaging
HAM-A: Hamilton Anxiety Rating Scale
HAM-D: Hamilton Depression Rating Scale
MMSE: Mini Mental State Examination
ReHO: regional homogeneity
SCD-Q9: Subjective Cognitive Decline Questionnaire–9

## Data Availability

The datasets used and/or analyzed during the current study are available from the corresponding author upon reasonable request. The trial data will be publicly available for publication in international peer-reviewed journals, regardless of whether the results are positive or negative.
